# Lanthanide Luminescence in Visible-Light-Promoted Photochemical Reactions

**DOI:** 10.3390/molecules25173892

**Published:** 2020-08-26

**Authors:** Ramiro Barraza, Matthew J. Allen

**Affiliations:** Department of Chemistry, Wayne State University, 5101 Cass Avenue, Detroit, MI 48202, USA; ramiro.barraza@wayne.edu

**Keywords:** catalysis, lanthanides, luminescence, photoluminescence, photoredox, visible light

## Abstract

The excitation of lanthanides with visible light to promote photochemical reactions has garnered interest in recent years. Lanthanides serve as initiators for photochemical reactions because they exhibit visible-light-promoted 4f→5d transitions that lead to emissive states with electrochemical potentials that are more negative than the corresponding ground states. The lanthanides that have shown the most promising characteristics for visible-light promoted photoredox are Sm^II^, Eu^II^, and Ce^III^. By understanding the effects that ligands have on the 5d orbitals of Sm^II^, Eu^II^, and Ce^III^, luminescence and reactivity can be rationally modulated using coordination chemistry. This review briefly overviews the photochemical reactivity of Sm^II^, Eu^II^, and Ce^III^ with visible light; the properties that influence the reactivity of these ions; and the research that has been reported towards modulating their photochemical-relevant properties using visible light and coordination chemistry.

## 1. Introduction

The ability to use visible light as a driving force for synthetic reactions is desirable in catalysis because it enables temporal control, minimization of side reactions relative to higher-energy light sources, and compatibility with modern synthetic techniques such as flow reactors [1,2,3,4,5,6]. Photochemical reactions typically involve radical processes, and often these radical processes can be used to install functional groups that would be otherwise difficult to install [7]. In the field of photocatalysis, transition-metal-mediated photoredox catalysis is a heavily studied area because of the efficiency and tunability of the photoredox-relevant properties of transition-metal complexes; metals such as Ru [1,2,3,4,6,8], Ir [1,2,3], and others [9] have been studied for their efficient photocatalytic properties. Properties used to define an effective photocatalyst include absorbance, excitation, and emission wavelengths; quantum yield; luminescence lifetime; and the ground- and excited-state electrochemical potentials of metal complexes [10].

The absorbance and excitation wavelengths of a complex are the first indicators of usefulness in visible-light-promoted reactions. Visible-light-promoted reactions are favorable over UV-promoted reactions because side reactions can be promoted by higher-energy UV light. Absorbance and excitation wavelengths outside of the visible-light range are not viable for visible-light-promoted reactions. The emission wavelength of a complex describes the energy of the emitted photons from a complex and also is related to the difference between ground- and excited-state electrochemical potentials. Quantum yield measures the efficiency of a complex to convert absorbed light into emitted light, where higher quantum yields indicate more efficient conversions. The luminescence lifetime of a complex is a measure of the time that a complex is in its excited state before emitting a photon. Longer luminescence lifetimes correlate with more opportunity for electron transfer from catalyst to substrate [10]. The ground- and excited-state electrochemical potentials of a complex describe the electrochemical properties of a complex and their thermodynamically allowed range of reactivity. The photochemical reactions discussed in this review are reductions; therefore, more negative excited-state potentials enable the reduction of compounds that are more difficult to reduce. If complexes are used catalytically, it is favorable to have ground-state electrochemical potentials that are fairly positive to enable facile turnover of catalytic cycles.

As the drive to develop new catalysts increases, focus has turned to Earth-abundant metals [7,9,11]. Among Earth-abundant metals are the lanthanides, which are orders of magnitude more abundant in the crust of the Earth than many transition metals commonly used in photoredox catalysis [12]. Lanthanides possess unique optical properties suitable for use in photochemical reactions because of their 4f→5d electronic transitions. The 4f valence orbitals of lanthanides are closer to the nucleus than the filled 6s and 5p orbitals. Because of the resulting shielding from the environment, electrons in 4f orbitals are not involved in covalent bonding with ligands. However, because the empty 5d orbitals are relatively diffuse, ligands interact with these orbitals and result in tunability of the luminescence of lanthanides via modulation of 4f→5d transitions.

**Figure 1:**
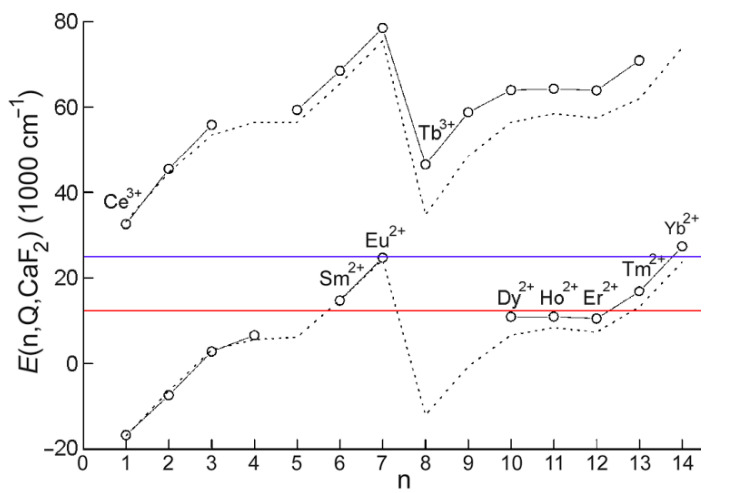
Energies of 4f→5d transitions for divalent and trivalent lanthanides in CaF_2_ versus the number of 4f electrons. Circles represent empirical data, and the dashed curve represents calculated values. The red and blue lines represent the boundaries of visible light. Lanthanides within or near that range are of interest because of their ability to be tuned into the visible-light range using coordination chemistry. From Dorenbos, P. FD transition energies of divalent lanthanides in inorganic compounds, *J. Phys: Condens. Matter* 2003, 15, 575–594. © IOP Publishing Ltd. Adapted with permission. All rights reserved [15].

For most trivalent lanthanides (the most common oxidation state of the lanthanides), 4f→5d transitions are too high in energy, typically in the UV or vacuum-UV region of the electromagnetic spectrum, to be useful for visible-light-promoted photochemical reactions [13,14]. Ce^III^ has the lowest energy 4f→5d transition (top lines in Figure 1) that can be tuned into the visible-light region using coordination chemistry [15]. Relative to the trivalent lanthanides, divalent lanthanides have low-energy 4f→5d transition energies (bottom lines in Figure 1). The 4f→5d transitions for divalent lanthanides range from the UV to the near-IR region of the electromagnetic spectrum. Because of the lower-energy 4f→5d transitions exhibited by divalent lanthanides and Ce^III^ compared to other oxidation states, divalent lanthanides and Ce^III^ are favorable to be used as catalysts for visible-light-promoted-photochemical reactions. Additionally, the luminescence properties of lanthanides capable of visible-light-promoted 4f→5d transitions are tunable because of the effect that ligands have on the 5d orbitals. In general, the tunability of the luminescence of lanthanides leads to interesting applications in photochemical reactions. It is worthy to note that lanthanides have a rich history of photochemical application [13,16,17,18,19,20,21,22,23]; however, recent studies demonstrated their viability in visible-light-promoted photochemical reactions. Beginning with the use of Sm^II^ in organic synthesis, visible-light-promoted lanthanide photoredox is a field dominated by a few lanthanides with promising reactivity. The three lanthanides that have been most studied for their visible-light-promoted photoredox properties are samarium, europium, and cerium.

Samarium is typically found in the +3 oxidation state; however, the +2 oxidation state of samarium is commonly used as a reducing agent in synthetic chemistry [24,25,26]. Sm^II^ has a ground-state electronic configuration of 4f⁶ and an excited-state electronic configuration of 4f^5^5d^1^. Samarium has a large crustal abundance, 4.7 ppm, compared to other metals commonly used in photochemical reactions, such as ruthenium and iridium that have crustal abundances of 0.34 and 0.022 ppb, respectively [12]. More than any other divalent lanthanide, divalent samarium has a rich history of use in synthetic chemistry because of its negative electrochemical potential and highly tunable reactivity that has been extensively studied in the reduction of organic compounds [24,25]. To reduce organic substrates with extremely negative electrochemical potentials, the use of additives, such as hexamethylphosphoramide or water, can be used to shift the electrochemical potential of SmI_2_ to even more negative values [26]. Europium is typically found in the +3 oxidation state; however, it is also commonly found in the +2 oxidation state. Divalent europium has a ground-state electronic configuration of 4f^7^ and an excited-state electronic configuration of 4f^6^5d^1^. Therefore, modulation of the 5d orbitals through ligand interactions leads to shifts in the absorbance and emission wavelengths of europium complexes. When comparing the relative crustal abundance of europium to the crustal abundance of commonly used metals in photoredox catalysis, europium has a crustal abundance of 1.0 ppm [12]. Cerium in the +3-oxidation state has a ground state electronic configuration of 4f^1^ and an excited state electronic configuration of 4f^0^5d^1^. Cerium has a relatively large crustal abundance, 63 ppm, greatly exceeding the abundance of metals commonly used in photoredox catalysts.

The purpose of this review is to illustrate the visible-light luminescence properties of lanthanides that relate to photochemical reactions. This review is structured to provide a brief overview of select properties of Sm^II^, Eu^II^, and Ce^III^ visible-light-promoted photoreductants and their use in photochemical applications. For ease of making comparisons, all electrochemical potentials in this review were converted to reference the normal hydrogen electrode (NHE). Readers are referred elsewhere for details regarding these elements as UV-promoted photoreductants [19,20,27,28], transition-metal-mediated photoredox catalysis in synthesis [1,2,3,4,5,6], chemiluminescence [29,30,31,32,33] and solid-state luminescence [13,15] of lanthanides, and luminescence and photochemistry of actinides [34,35,36,37].

## 2. Samarium

### 2.1. Luminescence of Samarium

To expand the uses of Sm^II^, the photoexcitation of SmI_2_ was studied as a way to decrease the electrochemical potential of SmI_2_ with and without the use of additives [16,38]. SmI_2_ in tetrahydrofuran has two absorbance peaks in the visible-light range with maxima at 565 and 617 nm that were attributed to 4f^6^→4f^5^5d^1^ transitions [38]. The maximum emission wavelength of SmI_2_ in tetrahydrofuran is 760 nm with an excited-state lifetime of 240 ns [39]. The reducing ability of visible light promoted SmI_2_ was evaluated by the reduction of carbon–halide bonds in a variety of organic chlorides. SmI_2_ reduces 1-chlorododecane under irradiation with visible light between 560 and 800 nm. This reaction was unsuccessful under irradiation with UV light, suggesting that visible light was necessary to promote the reduction. The discovery of the photoinduced reduction ability of SmI_2_ led to studies regarding the effect of ligands and additives on the luminescence of SmI_2_.

### 2.2. Effect of Additives on Luminscence of Samarium

**Figure 2:**
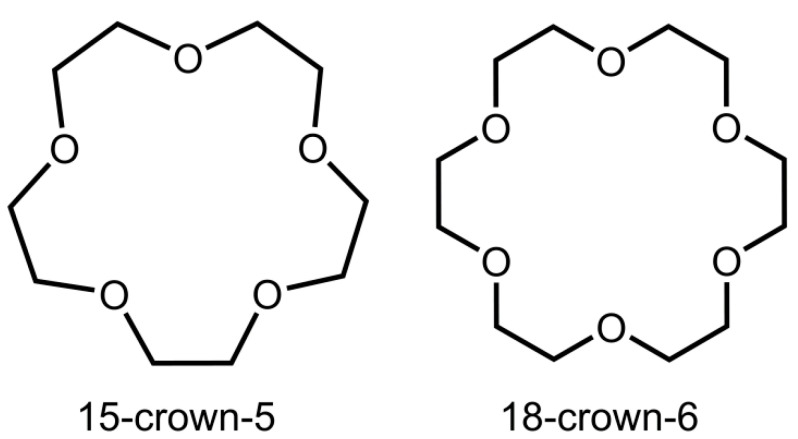
Ligands studied for their effects on the luminescence of Sm^II^ [41].

The reactivity of SmI_2_ is enhanced with the addition of coordinating solvents and additives, and this reactivity is typically influenced by coordination of the solvent or other additives—such as organic substrates, metal salts, or cosolvents—to Sm^II^ [24,25,26]. The luminescence of Sm^II^ involves 5d→4f transitions that are influenced by ligand environment; therefore, additives and ligands that coordinate to Sm^II^ can potentially alter the luminescence and reactivity of Sm^II^ [39,40]. Similar in effect to the nonphotoinduced reactivity of SmI_2_, hexamethylphosphoramide also enhances the photoinduced reactivity of SmI_2_ [40]. Mixtures of SmI_2_ and hexamethylphosphoramide have absorbance peaks at 580 and 619 nm, and the reactivity of the mixtures was assessed in the reduction of various aryl substrates (reactivity discusses in Section 2.3). To assess the effect of the ligands and solvent on the luminescence of Sm^II^, complexes of SmI_2_ and crown ethers were synthesized in tetrahydrofuran and acetonitrile [41]. The crown ethers that were assessed were 15-crown-5 and 18-crown-6 (Figure 2). Complexes of SmI_2_ with 15-crown-5 (**1**) or 18-crown-6 (**2**) were not soluble in tetrahydrofuran; however, these studies found that complexes **1** and **2** in acetonitrile underwent a color change during complexation. Complex **1** was made in a mixture of SmI_2_ and 2 equivalents 15-crown-5. Complex **2** was prepared in a mixture of SmI_2_ and 2 equivalents 18-crown-6. Complex **1** has a maximum absorbance wavelength of 540 nm and a maximum emission wavelength of 723 nm (Table 1). Complex **2** has a maximum absorbance wavelength of 606 nm and a maximum emission wavelength of 712 nm. The absorbance of complexes **1** and **2** were both hypsochromically shifted from the absorbance of SmI_2_, with complex **1** having a larger shift. In this study, SmI_2_ did not luminesce in acetonitrile, whereas complex **1** has a quantum yield of 0.30 ± 0.01 and complex **2** has a quantum yield of (4.0 ± 0.2) × 10^−4^. Complexes **1** and **2** have lifetimes of 0.80 ± 0.01 and 0.02 ± 0.01 µs, respectively. These studies also found that 15-crown-5 stabilizes SmI_2_ to atmospheric conditions, causing complex **1** to take ~24 h to oxidize in open atmosphere compared to near immediate oxidation of SmI_2_ in open atmosphere. One of the challenges in using divalent lanthanides for photoredox catalysis is oxidation to the trivalent oxidation state under open atmosphere. The discovery of the photoactivation of SmI_2_ and its use as a photoreductant [38] led to many investigations into the utility of SmI_2_ as a photoreductant for organic reactions, but there is still much left to understand about the effect of additives and ligands on the luminescence of Sm^II^.

### 2.3. Photochemical Applications of Samarium

**Scheme 1:**
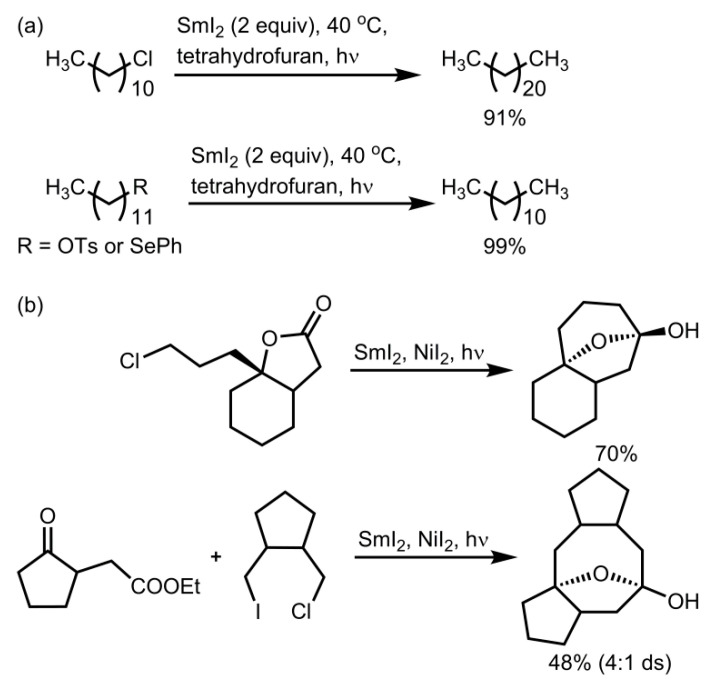
Photochemical reactions of Sm^II^ (**a**) with halides, tosylates, and chalcogenides and (**b**) in ring-closings [16,38,42].

Extensive use of SmI_2_ in organic reductions demonstrates that it is an excellent reductant; however, the use of Sm^II^ as a visible-light-promoted reductant is promising because it opens a pathway to strengthening the reducing power of Sm^II^. SmI_2_ in tetrahydrofuran has an electrochemical potential of −0.74 V vs. NHE [41]. SmI_2_ in tetrahydrofuran was initially studied as a visible-light-promoted photoreductant for the reduction and carbonylation of organic chlorides by Ogawa and coworkers [38]. They showed that SmI_2_ reduces a variety of organic chlorides in good yields when irradiated by light between 560 and 700 nm. SmI_2_ reduces organic chlorides in the presence of ketones, ethers, and aromatic groups. It also carbonylates primary and secondary alkyl chlorides in the presence of alkenes with yields between 61 and 89%. Ogawa and coworkers later demonstrated that photoexcited SmI_2_ reduces tosylates and chalcogenides (Scheme 1a) [16]. These initial studies regarding SmI_2_ as a visible-light-promoted photoreductant led to more studies into the scope of reactivity of Sm^II^. SmI_2_ in tetrahydrofuran was used to initiate intramolecular nucleophilic acyl substitutions to yield seven-, eight-, and nine-membered carbocycles (Scheme 1b) [42]. The use of visible-light-promoted SmI_2_ to initiate intramolecular nucleophilic acyl substitutions and form a seven-membered carbocycle was compared to the use of nonphotoinduced SmI_2_ and hexamethylphosphoramide for the same reduction. Visible-light-promoted SmI_2_ affords a greater yield (70%) compared to the yield of nonirradiated SmI_2_ (36%). Visible-light-promoted SmI_2_ also reduces 1,4-dihalides in the presence of a cyclic ketoester to yield a tricyclic 5:8:5 carbocycle, which is the framework of many natural products, in 48% yield (Scheme 1b). The visible light activation of SmI_2_ also enables SmI_2_ to reduce difficult-to-reduce substrates.

**Scheme 2:**
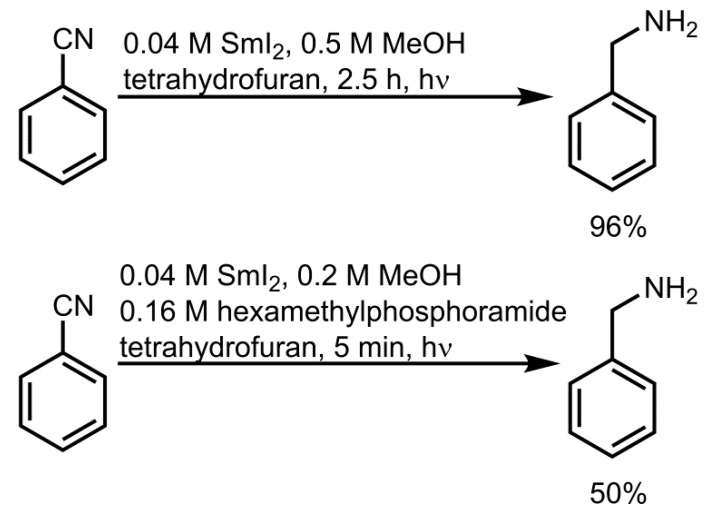
Reduction of cyanobenzene to benzylamine by visible-light-promoted SmI_2_ in the presence of methanol [43] and hexametylphosphoramide [40].

SmI_2_ also initiates the reduction of nitriles that are difficult to reduce [43]. In one example (Scheme 2), SmI_2_ reduces aromatic and aliphatic nitriles. In these studies, the reactions were performed in tetrahydrofuran; however, the addition of methanol is necessary for the reduction of nitriles. Methanol affords higher yields for the reductions by complexing to SmI_2_ and creating a partial solvation shell that assists SmI_2_ in capturing the radical anion from the reduced nitrile. Complexation of methanol, a proton source, to SmI_2_ is necessary for protonation of the radical anion. However, too much methanol results in diminished yields because methanol-filled coordination sites inhibit coordination of the nitrile to SmI_2_. Other studies also have explored the importance of additives to the electron transfer rate and reactivity of visible-light-promoted SmI_2_ reductions. One such additive is hexamethylphosphoramide, which showed an increase in yield of up to 20% for reduction of cyanobenzene when comparing the nonphotoinduced reaction to the photoinduced reaction [40]. This reactivity suggests that additives that improve the reactivity of nonphotoinduced SmI_2_ could potentially have the same effect in photoinduced systems of SmI_2_. For details on electron transfer and reaction rate, readers are referred elsewhere [44,45].

## 3. Europium

### 3.1. Luminescence of Europium

The luminescence of europium has been widely studied and used in many applications, such as optical electronic devices and fluorescent lamps [13,46]; however, the majority of these applications are based on the 4f→4f transitions of europium. These transitions are not favorable for applications in photochemical reactions because the shielded nature of the 4f orbitals does not associate these transitions with negative electrochemical potentials that are useful for light-promoted reduction chemistry. Because the luminescence of divalent europium is a result of electronic transitions between 4f and 5d orbitals, the tunability of the luminescence of divalent europium is derived from ligand interactions with its 5d orbitals. This section describes some of the effects that ligands have on the 4f and 5d orbitals of Eu^II^, and thus its luminescence, and describes examples of the reactivity of Eu^II^ in visible-light-promoted reactions.

#### Ligand Effects on Luminescence of Europium

**Figure 3:**
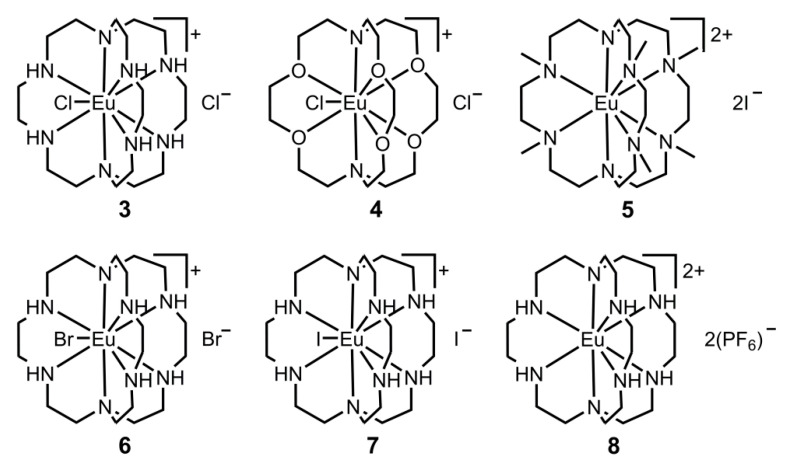
Complexes **3**–**8** studied for the effects of cryptands on the luminescent properties of Eu^II^ [47,55,59,60].

The tunabilty of the luminescence of divalent europium has been studied by altering the coordination environment of Eu^II^ with a variety of ligands and organometallic moieties, ranging from crown ethers [47] to sandwich complexes [48,49,50,51,52] to other divalent complexes [30,31,32,53,54]. However, the majority of these complexes do not absorb visible light. Recent studies focused on understanding ligand effects on the visible-light absorption and luminescence of divalent europium in discrete complexes in solution [55,56,57,58,59,60]. Some of these studies arose following the discovery of the brightly luminescent complex **3** (Figure 3) [55], which was later reported as the first europium-containing catalyst for visible-light-promoted photoreductions [57]. Complex **3** was synthesized by mixing a solution of EuCl_2_ with a solution of azacryptand 1,4,7,10,13,16,21,24-octaazabicyclo[8.8.8]hexacosane. Complex **3** was initially observed to luminesce yellow in basic water upon absorption of blue light [55]. The complex has a maximum absorbance at 415 nm and a maximum emission at 580 nm. In basic water, complex **3** has a quantum yield of 26%, which is the highest reported quantum yield of a divalent europium-containing complex in aqueous solution. Complex **3** was also studied in methanol, where it has a quantum yield of 37% [57]. This difference in quantum yield from an aqueous solution to methanol is attributed to the decrease in concentration of O–H oscillators upon moving from water to methanol, resulting in fewer O–H oscillators to quench luminescence. To understand why complex **3** absorbs visible light, it was compared to complex **4** and complex **5** that have similar structures to complex **3** but vary in donor atom identity and substitution of amines. Although complex **4** and complex **5** do not absorb visible light, the structural similarities among complexes **3**, **4**, and **5** enabled comparison of the three complexes to provide insight into the cause of visible-light absorption.

Complex **4** has two absorbance bands centered at 254 and 317 nm and a maximum emission wavelength of 468 nm. Complex **4** has a quantum yield of 9.3% and a lifetime of 0.2 µs [47]. Complex **5** has two absorbance bands with maxima at 261 and 345 nm and a maximum emission wavelength of 447 nm. Complex **5** has a quantum yield of 47%, which is one of the largest quantum yields for a discrete complex of divalent europium, and it has a lifetime of 1.25 µs [59]. The high quantum yield is due to both the lack of N–H oscillators and a shielding effect of the methyl groups. The methyl groups on the coordinating nitrogen atoms create a steric effect that prevents inner-sphere coordination of O–H oscillators.

**Figure 4:**
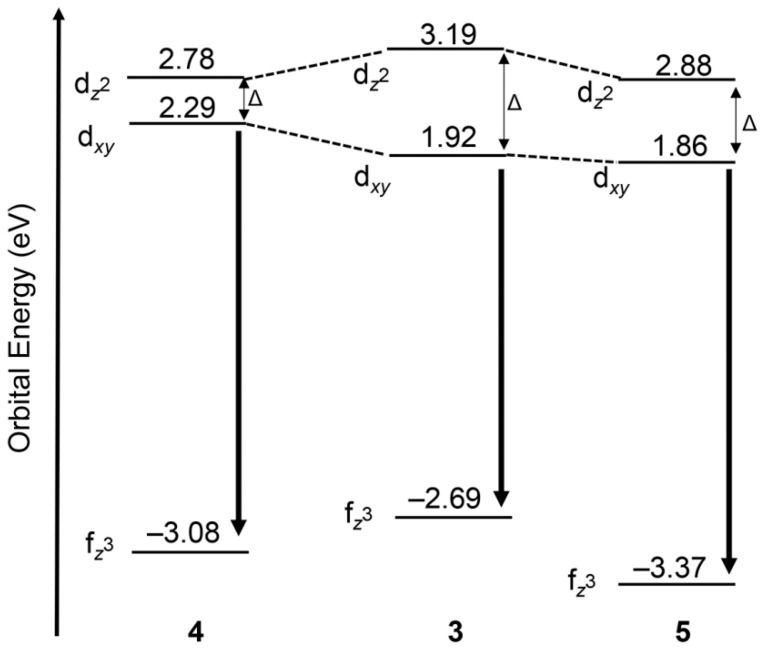
Orbital energy diagram of the 5d*_z_*^2^, 5d*_xy_*, and 4f*_z_*^3^ orbitals of europium-containing complexes **3**, **4**, and **5** [58,59].

To understand the effect of cryptands in complexes **3**–**5** on the 4f→5d transitions of europium, time-dependent density functional theory calculations of the absorbance and luminescence of complexes **3**–**5** were performed [58,59]. These calculations suggested that the absorbance at 415 nm for complex **3** was a transition between a 4f-type orbital and a 5d-type orbital. Calculations also revealed higher energy excitation at 260 nm for complex **3**. This transition was attributed to a transition between a 4f*_z_*^3^-type orbital and a 5d*_z_*^2^-type orbital. The calculations also revealed two excitations for complex **4**. First, an excitation at 259 nm was calculated to be a transition between the 4f*_z_*^3^-type orbital and the 5d*_z_*^2^-type orbital. The second excitation was a small shoulder at 365 nm that was calculated to be a 4f→5d*_xy_* transition. When comparing the orbital energies of complex **3** and complex **4**, it is notable that complex **3** has a larger d-orbital splitting (denoted as Δ) than complex **4** (Figure 4). This increase in d-orbital splitting enabled complex **3** to have a lower energy transition between its 4f and 5d orbitals. The difference in d-orbital splitting arises from the stronger-field character of coordinating nitrogen atoms in complex **3** compared to the coordinating oxygen atoms in **4**. Complex **3** also has a higher-energy 4f*_z_*^3^ orbital that further decreases the 4f→5d transition, enabling the transition to be promoted by visible light. The difference in 4f*_z_*^3^-orbital energy was attributed to the greater electronegativity of the oxygen, which had long range electrostatic effects that stabilized the 4f*_z_*^3^ in complex **4** [58].

Calculations for complex **5** revealed two absorbance bands centered at 268 and 357 nm, and a maximum emission wavelength of 384 nm attributed to 5d→4f transitions [59]. The transition at 357 nm was calculated to be between a 4f-type orbital and a 5d*_xy_*-type orbital. Considering the difference in donor strength and spectroscopic properties between complex **3** and complex **4**, complex **5** was expected to display a bathochromic shift in spectroscopic properties compared to complex **3**. However, complex **5** had a smaller d-orbital splitting, and a lower energy f*_z_*^3^. The differences in the d-orbital splitting and the lower energy 4f-orbital were due to the difference in geometry. Complex **3** has a distorted hula-hoop geometry, but complex **5** is a distorted bicapped trigonal antiprism. To make a direct comparison based solely on donor strength, time-dependent density functional theory calculations were performed with complex **3** locked into a distorted bicapped trigonal antiprism. These calculations suggested that complex **3**, while locked into the same geometry as complex **5**, had a smaller d-orbital splitting, consistent with expectations. This computational experiment supported the premise that atoms with greater strong-field character will have greater d-orbital splitting; however, these studies also indicated that geometry and coordination number of a ligand have a greater effect on the luminescence of europium [59].

To study the effect of the ninth coordination site of complex **3**, the effect of various counterions (Cl^−^, Br^−^, I^−^, and PF_6_^−^) and solvents (methanol and acetonitrile) on the luminescence of complex **3** was explored (Table 2) [60]. The luminescence properties of complexes **3** and **6**–**8** in methanol were similar to each other with maximum absorbance values between 402 and 405 nm and identical maximum emissions of 577 nm. This lack of difference among the complexes in methanol suggests that either methanol fills the ninth coordination site in each complex or that complexes **3** and **6**–**8** are eight coordinate. To further understand the effect of each counteranion, the aforementioned complexes were studied in acetonitrile, which is less likely than methanol to displace coordinated halides. Complexes **3** and **6**–**8** in acetonitrile showed a larger dependence on the identity of the counteranion than complexes **3** and **6**–**8** in methanol. Complexes **3** and **6**–**8** in acetonitrile have absorption maxima that range between 380 and 405 nm. The absorption of each complex exhibits a blue shift from PF_6_^−^ and I^−^ to Br^−^ to Cl^−^. A similar trend is also exhibited by the emission of complexes **3** and **6**–**8** in acetonitrile. Complexes **8** and **9** in acetonitrile have an emission of 577 nm, which indicates that I^−^ and PF_6_^−^ are not coordinating to the cryptate, but instead acetonitrile is coordinating or the complexes are eight coordinate in solution [60]. This study provides insight on the effects of solvent and counteranion of the luminescence of Eu^II^-containing ternary cryptates in solution that is valuable towards the fine-tuning of the luminescence properties of Eu^II^-containing complexes.

**Figure 5:**
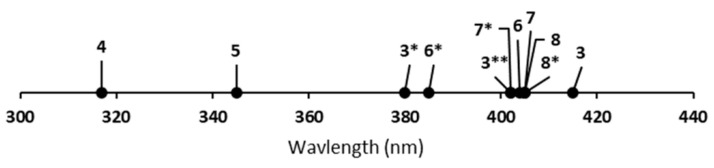
Comparison of the maximum of absorbance bands of complexes containing Eu^II^ in solution in methanol [57,59,60]. * Complexes in acetonitrile. ** from [60].

**Figure 6:**
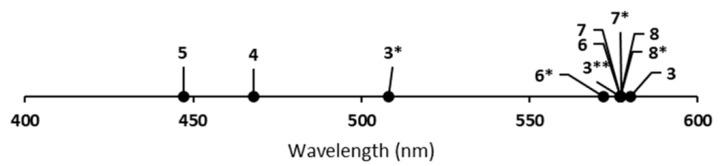
Comparison of the maximum emission wavelengths of complexes containing Eu^II^ in solution in methanol [57,59,60]. * Complexes in acetonitrile ** from [60].

The Eu^II^ studies described so far in this review demonstrate that by changing ligand environment, the visible-light promoted luminescence of Eu^II^ shifts significantly, and these results parallel observations in the UV using Eu^II^ and crown ethers and cryptands [47,55,59]. Figure 5 and Figure 6 summarize the shift in absorbance and luminescence among each Eu^II^-containing complex discussed in this section. Complexes in methanol had red-shifted absorbances compared to EuCl_2_, almost regardless of counteranion. Complexes in acetonitrile with large counteranions have redshifted absorbance and emission wavelengths. Complexes with smaller counteranions, that are more likely to be coordinated to Eu^II^, have slightly more varied absorbance wavelength and similar emission wavelengths. The studies suggest that complexes of Eu^II^ in acetonitrile have the broadest range of tunability among the mentioned complexes. However, complexes of Eu^II^ in methanol yield more red-shifted absorbances than complexes of Eu^II^ in acetonitrile regardless of the counterion. According to this trend of complexes in acetonitrile and methanol, complexes in solvents that are less likely to displace coordinated anions have a broader range of tunability. This tunability is directed by the identity of the counteranion and has less of an effect from coordinated solvent molecules. Complex **4** had the most blue-shifted absorbance, demonstrating that Eu^II^-containing complexes with ethereal donors have blue shifted absorbance wavelengths when compared to complexes with nitrogenous donors. Complex **5** has a blue-shifted absorbance and emission relative to complex **4**; however, when comparing these two complexes, the ligand affects the overall geometry of the complex. These studies provide insight into the properties that affect the luminescence of Eu^II^-containing complexes to aid in future ligand design and demonstrate that there remains a wealth of knowledge to understand about the tuning of Eu^II^ luminescence.

### 3.2. Photochemical Applications of Europium

The visible-light absorbance and long-lived excited state of complex **3** made it a promising candidate for photoredox catalysis. To predict the range of useful reactivity of complex **3**, the Rehm–Weller formalism was used. The Rehm–Weller formalism takes into consideration the ground-state potential (*E*_1/2_) and the energy of the emissive state (*E*_0,0_) to estimate the excited-state electrochemical potential (*E*_1/2_*) of a complex [61,62]. The *E*_1/2_ of 2+/3+ couple of complex **3** was determined via cyclic voltammetry to be −0.70 V vs. NHE [55]. That ground-state potential, together with the visible-light emission, resulted in an estimation of the excited-state electrochemical potential (*E*_1/2_*) to be −2.8 V vs. NHE. The reactivity of complex **3** was evaluated by testing its reactivity for the reduction of organic chlorides with reduction potentials near the estimated excited-state electrochemical potential [57]. Reduction potentials of substrates varied from −2.14 to −2.85 V vs. NHE. The estimated excited-state electrochemical potential of complex **3** is more negative than the reduction potentials of 1-chlorobenzene (**10**), allylchloride (**11**), and benzyl chloride (**12**) (Table 3). Because of this difference, the excited-state of complex **3** is thermodynamically sufficient to reduce substrates **10**–**12**. Substrates **11** and **12** had the best yields of reductive coupling, which could partially be due to the volatility of the reduction products of the other substrates. Although *tert*-butyl chloride (**9**) had a slightly (0.05 V) more negative reduction potential than that of complex **3**, complex **3** was able to reduce the substrate.

The reduction of substrate **12** by complex **3** was studied stoichiometrically and catalytically. For the stoichiometric reaction, EuCl_2_, azacryptand, and **12** were stirred at a 1:1:1 ratio under blue light to yield 85 ± 2% of the coupled product within 30 min [57]. Control experiments without ligand, without EuCl_2_, and without light were conducted and showed no reactivity unless all three components were in the reaction mixture. The catalytic reaction was examined by stirring 10 mol % of EuCl_3_ and azacryptand with substrate **12** and Zn^0^ as a sacrificial reducing agent. This reaction had an 80 ± 10% yield of 1,2-diphenylethane and 11 ± 2% toluene formation, where toluene is the uncoupled reduction production of substrate **12**. Variations in catalytic loading were also examined. A catalytic loading of 5 mol % yielded 71% of the coupled product and 12% toluene formation (~14 turnovers); a catalytic loading of 1 mol % yielded 70% coupled product and 21% toluene formation (~70 turnovers); and a loading of 0.5 mol % had a 60% yield of coupled product and 26% toluene formation (~120 turnovers). Further decrease in catalytic loading to 0.005 mol % yielded only toluene. These results indicate that decreased loading increases toluene formation likely due to decreased amounts of benzyl radical available for coupling with less catalyst.

## 4. Cerium

### 4.1. Luminescence of Cerium

As with divalent lanthanides, much of the tunability of luminescence relevant to photochemical reactions of Ce^III^-containing complexes originates from ligand interactions with 5d orbitals to influence 4f→5d transition energies. The 5d orbitals of cerium are important because Ce^III^ readily undergoes tunable 4f→5d transitions, along with 4f→4f transitions that are commonly observed in trivalent lanthanides. This distinction arises in large part because of its accessible tetravalent oxidation state [13]. By varying ligand identity, 4f→5d transitions can be tuned to enable different absorbance and emission wavelengths, including visible light. This section describes some effects that ligands have on the luminescence of Ce^III^ visible light promoted photocatalysts. For more information on luminescence of nonphotoreducing Ce^III^ complexes and reactivity of Ce^III^ outside of the visible light range, readers are referred elsewhere [27,28,63].

#### Ligand Effects on Luminescence of Cerium

**Figure 7:**
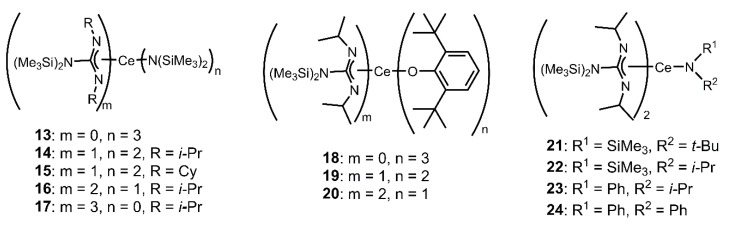
Guanidinate, amido, and aryloxide complexes **13**–**24** studied with respect to the luminescence of Ce^III^ [64,65,66].

There have been many studies that have focused on the luminescence properties of cerium for lighting and other applications [13,22]. Recent studies describing the modulation of cerium luminescence for visible-light-promoted photoreductions have been reported by Schelter and coworkers [7]. The first cerium guanidinate complexes that they reported for photocatalysis were monoguanidinate complexes derived from cerium tris(bis(trimethylsilyl)amide) (**13**) [64]. Complexes **13**–**15** (Figure 7) were synthesized to explore the effect of functionalization of the guanidinate ligand on the luminescence of cerium. Complex **13**, without a di-imide, has weak yellow emission. Insertion of a di-imide results in complexes **14** and **15** that display bright green emission. Investigation into the absorbance and luminescence of each complex revealed that although all three complexes have similar absorbance wavelengths, the emission wavelengths of **14** and **15** are hypsochromically shifted about 30 nm from complex **13**. Complexes **14** and **15** also have large quantum yields, 46 and 53%, respectively, compared to complex **13**, which has a quantum yield of 3%. Furthermore, complexes **14** and **15** have long lifetimes, 67 and 61 ns, respectively, compared to complex **13**, which has a lifetime of 24 ns. Time-dependent density functional theory calculations of the absorbance and luminescence of complexes **14** and **15** revealed that the lowest-energy transition for both complexes is between a ground-state orbital with 4f character and an excited-state orbital with 5d*_z_*^2^-type character [64]. These calculations reinforced the importance of modulating the energy of the 5d orbital with ligands. The luminescence studies demonstrated that complexes **14** and **15** have larger quantum yields and longer lifetimes than complex **13**; however, complex **13** is the best catalyst for the coupling of benzyl chloride (reactivity is discussed in Section 4.2).

To understand how the complexation of guanidinate ligands effects the luminescence of cerium, Schelter and coworkers studied a series of guanidinate and amide cerium complexes (**13**, **14**, **16**, and **17**) that complete the series of mixed guanidinate and amide cerium complexes [65]: complex **13** contains all bis(trimethylsilyl)amido ligands; complex **14** has one di-isopropyl guanidinate ligand and two bis(trimethylsilyl)amido ligands; complex **16** has two di-isopropyl guanidinate ligands and one bis(trimethylsilyl)amido ligand; and complex **17** has three di-isopropyl guanidinate ligands. Complexes **13**, **14**, **16**, and **17** all have maximum absorbance wavelengths of 420 nm, and time-dependent density functional theory studies suggested that these absorbances are attributed to transitions between ground-state orbitals with 4f character and excited-state orbitals with 5d character. The acceptor orbitals of the 4f→5d transitions for complexes **13** and **17** are degenerate 5d*_z_*^2^-type orbitals as suggested by natural transition orbital calculations. The 4f→5d transition acceptor orbitals for complexes **14** and **16** were calculated to be either 5d*_xz_*- or 5d*_xy_*-type orbitals. Although complexes **13**, **14**, **16**, and **17** have maximum absorbance wavelengths at 420 nm, they have a wide range of Stokes shifts (from 35 to 135 nm) that decrease with increasing number of guanidinate ligands. Additionally, complexes **13**, **14**, **16**, and **17** have long-lived excited states with lifetimes of 24, 65, 117, and 83 ns, respectively [65]. Long lifetimes indicate that little energy is lost through nonradiative decay, and the presence of guanidinate ligands decreases the amount of energy lost through nonradiative decay by ligand vibrational modes.

Cerium complexes of aryloxide ligands **18**–**20** were also synthesized and studied for their luminescence properties [65]. Complexes **18**–**20** differ in the ratio of di-isopropyl guanidinate and di-*tert*-butyl aryloxide ligands. Complex **18** is a cerium complex with three coordinating aryloxide ligands. Similar to the observations from complexes **13**, **14**, **16**, and **17**, complexes **18**–**20** have maximum absorbance wavelengths of 420 nm and vary in maximum emission wavelengths with Stokes shifts from 39 to 112 nm. Quenching studies revealed that complexes with bis(trimethylsilyl)amido ligands have lower quantum yields and larger Stokes shifts because of quenching effects from ligand vibrational modes. Replacement of amido ligands with aryloxide or guanidinate ligands suppresses these quenching effects. Consequently, complexes with fewer amido ligands have smaller Stokes shifts and larger quantum yields. The ligand vibrational modes are suppressed in aryloxide or guanidinate ligands because of their rigidity compared to amido ligands.

The Schelter group also studied the effect of amido ligand rigidity on the luminescence of bis(guanidinate) amido cerium complexes by varying the cone angle of amido ligands [66]. Bis(guanidinate) amido cerium complexes **16** and **21**–**24** (Figure 7) that have various cone angles were used in this study. The series of complexes **16** and **21**–**24** was split into two categories: category A, consisting of complexes **16** and **21** with cone angles of approximately 173°, and category B, consisting of complexes **22**–**24** with cone angles <156°. Complexes **16**, **21**, and **22** have two absorbance bands, and complexes **23** and **24** have one absorbance band. Complexes **16**, **21**, and **22** all have absorbance bands at approximately 350 nm that were calculated to arise from 4f→5d*_xz_* or 4f→5d*_yz_* transitions. The second absorbance band for complexes **16**, **21**, and **22** vary for each complex: 429 nm for complex **16**; 440 nm for complex **21**; and 463 nm for complex **22**. The second absorbance bands were attributed to 4f→5d*_z_*^2^ transitions. The absorbance bands at 460 nm of complexes **23** and **24** were also attributed to 4f→5d*_z_*^2^ transitions. Quantum yield measurements of complexes **16** and **21**–**24** revealed that quantum yields decrease in the order **16** > **21** > **22** > **23 > 24** with quantum yields of 0.79, 0.75, 0.23, 0.14, and 0.10, respectively. In this series of complexes, **16** and **21**–**24**, quantum yields increase with increasing ligand rigidity. In the case of ligands **16** and **21**–**24**, ligand rigidity is dictated by the cone angle between the substituents of the amido ligand. The existence of ligand vibrational modes also decreased the lifetime of complexes **16** and **21**–**24**. Complexes **16** and **21**–**24** have lifetimes of 117, 221, 158, 41, and 43 ns, respectively [66]. The difference in lifetimes was attributed to a decrease in C–H bond vibrational modes from isopropyl to *tert*-butyl to trimethylsilyl functional groups. Lifetimes increase from complex **23** to **24** and follow the trend that decreasing the number of vibrational modes of ligands results in longer lifetimes. The following paragraphs discuss overall trends in the absorbance, luminescence, and electrochemical potentials of Ce^III^.

**Figure 8:**
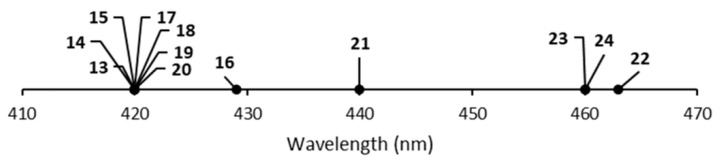
Comparison of the maximum of absorbance bands of Ce^III^-containing complexes **13**–**24** [64,65,66].

The overall trend in absorbance across the ligands discussed so far in this review demonstrates that Ce^III^-containing complexes with lower-energy absorbance wavelengths contain both guanidinate and amido ligands (Figure 8) [64,65,66]. Complexes **21**–**24**, with two guanidinate ligands and one amido ligand, are bathochromically shifted from complex **12**. Complexes **13**–**15** and **17**–**20** have maximum absorbances at 420 nm. The complexes vary from containing all amido ligands to mixed amido, aryloxide, and guanidinate ligands. This trend in absorbance suggests that the necessary scaffold for shortening the absorbance wavelength of complexes requires a bis(guanidinate)monoamido backbone. Further modulation of the absorbance of Ce^III^ was a result of varying the cone angle of substituents of the amido ligand [66].

**Figure 9:**
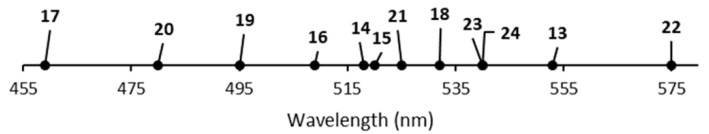
Comparison of the maximum emission wavelength of cerium complexes **13**–**24** [64,65,66].

Ligand modification affects the emission of cerium more than its absorbance. Whereas for absorbance, the largest difference in wavelength is between complexes **13** and **22** (43 nm), the largest difference in emission is between complexes **17** and **22** (116 nm) (Figure 9) [64,65,66]. Complex **17**, with three di-isopropyl guanidinate ligands, has the shortest emission wavelength at 459 nm. Overall, complexes with two guanidinate ligands and one amido ligand have the most bathochromically shifted emission wavelengths. However, complex **22** of this type has a larger emission wavelength than complex **13**, which is a Ce^III^ tris(trimethylsilyl)amido complex.

These studies highlight the wide range of tunability of the luminescence properties of Ce^III^-containing photoredox catalysts. By understanding which ligand properties influence the luminescence of Ce^III^, future Ce^III^-containing photoredox catalysts can be synthesized in a directed approach to achieve desirable properties. Based on the trends mentioned in this section, low energy absorbance wavelengths are achieved when a Ce^III^ complex contains both guanidinate and amido ligands and that the wavelength can be tuned further by changing the cone angle of the substituents on the amido ligand. Overall, the emission of Ce^III^-containing photoredox catalysts is shown to be most affected by changing the coordinating ligands. Similar to the trends seen in the absorbance wavelengths of complexes **13**–**24**, complexes that contain two guanidinate ligands and one amido ligand have longer emission wavelengths. By employing these trends, the synthesis of Ce^III^-containing photoredox catalysts has the potential to be streamlined to yield complexes with favorable photoredox properties.

### 4.2. Photochemical Applications of Cerium

**Figure 10:**
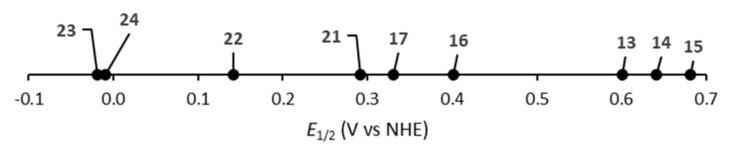
Comparison of the ground-state electrochemical potentials (*E*_1/2_) of cerium complexes **13**–**17** and **21**–**24** [64,65,66].

**Figure 11:**
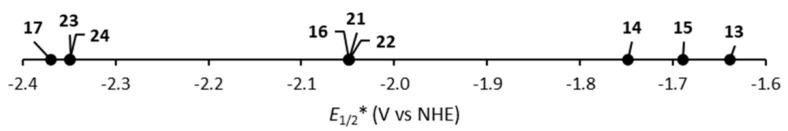
Comparison of the calculated excited-state electrochemical potentials (*E*_1/2_*) of complexes **13**–**17** and **21**–**24** [64,65,66].

In addition to studying the effects of ligands on the luminescence of Ce^III^, their effects on the reactivity of cerium in visible-light-promoted photoreactions were also evaluated [64,65,66]. The electrochemical potential of each complex was studied to evaluate reducing abilities. The electrochemical potentials of complexes **13**–**17** and **21**–**24** were determined using cyclic voltammetry (Figure 10), and the excited-state electrochemical potentials of complexes **13**–**17** and **21**–**24** were calculated (Figure 11) using the Rehm–Weller formalism. The excited-state electrochemical potentials of complexes **13**–**17** and **21**–**24** are notable because they are all more negative than the excited-state reduction potential of Ru(bpy)₃, which is one of the most used photoredox catalysts.

**Scheme 3:**
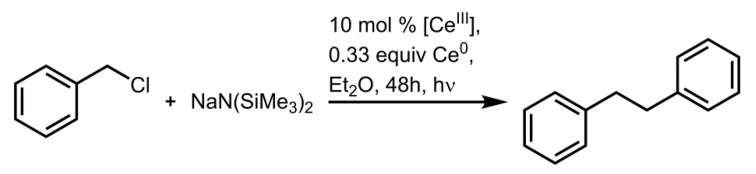
Catalytic reductive coupling of benzyl chloride using Ce^III^ [64].

**Scheme 4:**
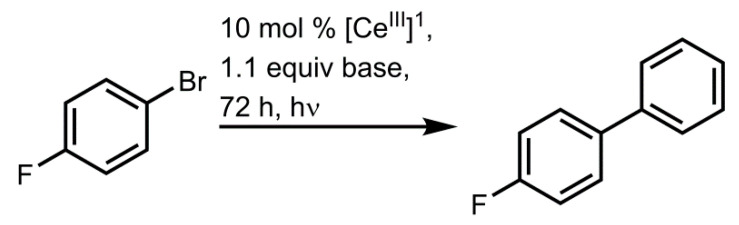
Arylation of 4-bromofluorobenzene promoted by Ce^III^. ^1^ 20 mol % for complex **13** [64,65,66].

Complexes **13**–**15** were studied for their ability to perform a benzyl chloride coupling (Scheme 3) and arylation of benzene (Scheme 4) [64]. To afford the reductive coupling of benzyl chloride, benzyl chloride was reacted with complexes **13**–**15** in a solution of diethyl ether containing sodium bis(trimethylsilyl) amide and elemental cerium powder. The presence of sodium bis(trimethylsilyl)- amide was necessary to reduce Ce^IV^ to Ce^III^, and elemental cerium is necessary to quench the resulting aminyl radical and prevent the production of byproducts. Complex **13** produces the highest yield of 1,2-diphenylethane (68%). Complexes **14** and **15** produce yields of 17 and 10%, respectively. The lower yields from complexes **14** and **15** were attributed to the steric bulk of the guanidinate ligands reducing the ability of benzyl chloride to coordinate Ce^III^ [64].

Complexes **13**, **16**, and **21**–**24** were studied for their ability to catalyze the arylation of 4-bromofluorobenzene (Scheme 4) [64,65,66]. Each complex was reacted with 4-bromofluorobenze in a solution in benzene containing a base, which corresponded to the amido group of each complex, as a sacrificial reducing agent. Complex **13** was reacted with 4-bromofluorobenzene and sodium bis(trimethylsilyl)amide to yield 43% of 1-(4-fluorophenyl)benzene [64]. Reactions of 4-bromo-fluorobenze with complexes **16**, **21**, **22**, **23**, and **24** resulted in yields of 0, <5, 12, 36, and 66%, respectively. Only complexes with a cone angle less than 156° were able to promote the coupling [66]. Overall, complexes with larger cone angles resulted in higher yields of the arylated product. The lack of reactivity from complexes **16** and **21**, which have cone angles larger than 170°, suggests that complexes **16** and **21** are too sterically bulky to allow interaction of Ce^III^ and 4-bromofluorobenzene. These studies suggest that when designing visible light promoted Ce^III^ catalysts, the steric bulk of coordinating ligands must be taken into consideration as to enable substrates to be able to coordinate to Ce^III^.

## 5. Other Lanthanides

**Figure 12:**
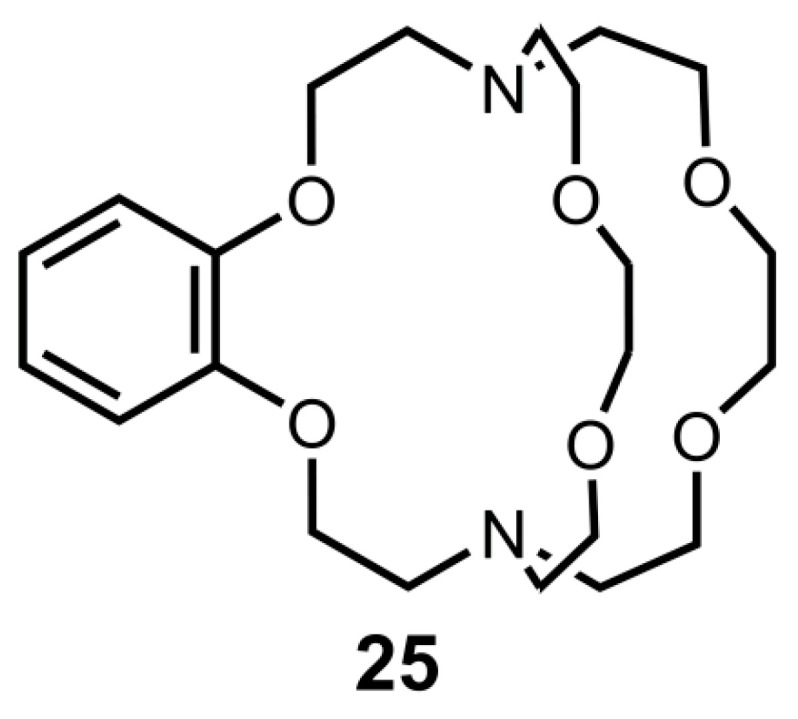
Cryptand **25** which was studied for its effect on the luminescence properties of Yb^II^ [64].

The most thoroughly studied lanthanides with respect to luminescence in visible-light-promoted photochemical reactions have been Sm^II^, Eu^II^, and Ce^III^; however, the rest of the lanthanide series has been recently isolated in the divalent oxidation state [67,68]. Because of the negative reduction potentials [69] and the tunability of luminescence properties by coordination environment, divalent lanthanides have great potential to be studied further in visible-light-promoted photoredox catalysis. One example of these elements is Yb^II^. YbI_2_, which has an electrochemical potential (*E*_1/2_) of −1.15 V vs. NHE, has been shown to reduce alkyl halides, tosylates, and chalcogenides when irradiated by UV light [16]. These reactions are irradiated with UV light; however, recent studies demonstrate that Yb^II^ can be complexed with cryptands to yield favorable luminesce properties for visible-light-promoted photoreductions. One such study compares the tunabilty of the luminescence and electrochemical properties of Yb^II^ with comparison to analogous Eu^II^ complexes [70]. YbI_2_ has a maximum absorbance wavelength of 368 nm and a maximum emission wavelength of 423 nm. Like the other divalent lanthanides mentioned in this review, the absorbance and emission of Yb^II^ can be modulated by ligand environment. YbI_2_ was complexed with the cryptands used in complexes **3**–**5** (Figure 3) and ligand **25** (Figure 12). The resulting Yb^II^-containing complexes were studied for their luminescence [70]. Overall, these Yb^II^-containing complexes displayed similar trends to their Eu^II^-containing analogs in their maximum absorbance wavelengths, where stronger field donors have a larger effect in bathochromically shifting absorbance; however, their maximum emission wavelengths showed that only the Yb^II^-containing analog of **3** bathochromically shifted the emission of YbI_2_. This study compared the effects of cryptands on the luminescence and electrochemical properties of Yb^II^ and Eu^II^ to observe the similarities and differences among two divalent lanthanides. Further studies such as these could open the way to developing directed ligand design for desirable properties of Yb^II^ and other divalent lanthanides for use in visible-light-promoted photoredox catalysis. Recently, the visible-light-promoted photocatalytic activity of complexes of trivalent gadolinium, lutetium, and lanthanum were reported [11]. Gd^III^, Lu^III^, and La^III^ were complexed to pentadentate expanded porphyrins called texaphryins. These complexes of Gd^III^, Lu^III^, and La^III^ facilitate the stabilization and oxidation of curcumin, a natural product with medicinal properties, to produce other compounds of medicinal interest [11].

## 6. Conclusions

The study of lanthanides in visible-light-promoted photoredox catalysis has expanded past samarium in recent years. Initial studies into other lanthanides, such as cerium and europium, are proving to be exciting and leave new questions to be answered. Furthermore, the recent report of all of the lanthanides (except Pm) in the divalent oxidation state combined with their potential to be tuned into absorbing visible light (Figure 1) raises the possibility for the use of other divalent lanthanides with electrochemical potentials more negative than Eu^II^, Sm^II^, and Ce^III^ in visible-light-promoted photoredox reactions [67,68,69]. Further studies into ligand effects on 4f→5d transitions of the divalent lanthanides has the potential to yield photoredox catalysts with greater tunability and extremely negative electrochemical potentials.

## Figures and Tables

**Table 1:** Luminescence and electrochemical properties of SmI_2_, **1**, and **2** in acetonitrile [41].

Complex	Crown	*λ*_ab_ (nm)	*λ*_em_ (nm)	*E*_1/2_ (V vs. NHE)
**SmI_2_**	none	685	*	−0.74 ± 0.05
**1**	15-crown-5	540	723	−0.46 ± 0.05
**2**	18-crown-6	606	712	−0.77 ± 0.02

* SmI_2_ does not luminesce in acetonitrile.

**Table 2:** Maximum absorbance and emission wavelengths of complexes **3** and **6**–**8** in methanol and acetonitrile [60].

Complex	Solvent	*λ*_ab_ (nm)	*λ*_em_ (nm)
**3**	Methanol	402	577
**6**	Methanol	404	577
**7**	Methanol	405	577
**8**	Methanol	405	577
**3**	Acetonitrile	380	508
**6**	Acetonitrile	385	572
**7**	Acetonitrile	402	577
**8**	Acetonitrile	405	577

**Table 3:** Stoichiometric reduction of substrates **9**–**12** by complex **3** [57].

Compound Number	Substrate	*E*_pc_ (V vs. NHE)	Yield of Reduction (%)	Product of Reduction
**9**	*t*-BuCl	−2.85	1.9 ± 0.1	(*t*-Bu)_2_
**10**	PhCl	−2.73	5.4 ± 0.4	C_6_H_6_
**11**	CH_2_CHCH_2_Cl	−2.15	46 ± 2	(CH_2_CHCH_2_)_2_
**12**	BnCl	−2.14	85 ± 2	Bn_2_
